# 

**Published:** 1815-01

**Authors:** 


THE LONDON

### MEDICAL AND PHYSICAL

### JOURNAL.

CONDUCTED BY

SAMUEL FOTHERGILL, M.D.

PHYSICIAN EXTRAORDINARY TO HIS ROYAL HIGHNESS THE DUKE OF KENT; PHYSICIAN TO THE ASYLUM FOR FEMALE ORPHANS; AND TO THE WESTMINSTER GENERAL, AND THE WESTERN, DISPENSARIES.

VOL. XXXIII.

FROM JANUARY TO JUNE, 1815.

Et quoniam variant morbi, variabimus artes;

Mille mali species, mille salutis erunt.

5-134

*LONDON:*

PRINTED FOR THE PROPRIETORS, BY J. ADLARD, 23, BARTHOLOMEW-CLOSE, AND 39, DUKE-STREET, SMITHFIELD; AND PUBLISHED BY J. SOUTER, NO. 1, PATERNOSTER-ROW.

1815.

W1
L031

[Entered at Stationers' Hall.]

THE

### Medical and Physical Journal.

1 OF VOL. XXXIII.] JANUARY, 1815. [NO. 191.

"For many fortunate discoveries in medicine, and for the detection of nume-
"rous errors, the world is indebted to the rapid circulation of Monthly
"Journals; and there never existed any work to which the faculty in
"EUROPE and AMERICA were under deeper obligations than to the
"Medical and Physical Journal of London, now forming a long, but an
"invaluable series."—RUSH.

### ADDRESS.

*THE Proprietors of the* MEDICAL AND PHYSICAL JOURNAL *wish not to appear ungrateful for the encouragement they have received by its increased circulation. But the first tribute of gratitude is due to their numerous correspondents, whose contributions are now so considerable as to require some management in showing impartiality to each. They have one remark to make—that communications with the name of the writer will always claim a priority, and that even these will be distinguished as the subject may be of a temporary or transient nature. By attention to the latter, their Journal has now become a register for events which are partly forgotten, by fresh ones which arise, more interesting from their novelty. All, however, are important in a science which can only be perfected by constant observation, and by an accumulation of such facts as are with difficulty preserved, excepting in monthly archives, because the interest that attends them is often lost by a longer delay.*

*The editorship has never been considered a department in which economy should have any influence. To select good articles, to arrange them carefully, and even to superintend the press, is not the province of the proprietors; but, by taking the impartial advice of the most respectable part of the London Faculty, they cannot be long mistaken, and as often as they are, the same friendly assistance is always ready to direct them. To preserve a necessary uniformity in their Journal, they have been advised for the present to insert the name of only a single Editor, who has a discretionary power to avail himself of whatever assistance he may require in the various departments.*

*Some of our readers have remarked, that the Journal is less complete as a register, by the partial omission of some articles in a periodical work, which it was formerly our custom to compress into our review. We were often complimented for our impartiality to a rival Journal, and, if we find it the general wish of our readers, shall resume our former plan. It may not be amiss on this occasion to repeat, that the Editorial has always been distinct from the Reviewing Department. Though the Editors have not shrunk from a certain degree of responsibility in whatever has appeared under their name, yet it is with pleasure they remark, that complaints of unjust severity have never, that they know of, been imputed to this Journal. They have now authority to add, that whenever an author or his friends think his work unfairly treated, his answer shall always be inserted, without any remarks which may lessen the spirit of the defence, or interrupt the attention of the reader.*

### MONTHLY CATALOGUE OF MEDICAL BOOKS.

AN Illustration of Mr. Hunter's Doctrine particularly concerning the Life of the Blood, in answer to the Edinburgh Review of Mr. Abernethy's Lectures. By Joseph Adams, M.D. Author of Observations on Morbid Poisons. 8vo.—Callow.

Report of the Medicinal Effects of an Aluminous Chalybeate Spring lately discovered at Sandrocks, in the Parish of Chale, in the Isle of Wight; pointing out its Efficacy in the Walcheren and other Diseases incident to Soldiers who have served abroad, &c. &c. By William Lempriere, M.D. 8vo.—Callow.

---

### *Recent American Publications.*

Experiments on the Production of Animal Heat by Respiration. An Inaugural Dissertation, read and defended at the public examination before the Rev. President and the Medical Professors of Harvard University, August 20, 1813. By Enoch Hale, jun. M.D. Cummings and Hilliard, Boston.

A Treatise on the Membranes in general, and different Membranes in particular. By Xavier Bichat, Physician of the Hotel Dieu, &c. with an Historical Notice of the Life of the Author, by M. Huson. Translated by John G. Coffin, M.D. 1 vol. 8vo.—Cummings and Hilliard, Boston.

Dissertation on the Natural History and Medieinal Properties of Ergot, delivered at the annual meeting of the Massachusetts Medical Society, in June, 1813. By Dr. Oliver Prescot. Published by the Society. Boston.

An Inaugural Dissertation on the Medical Properties of Gold. By John C. Cheeseman. New York, 1812.

An Eulogium to the Memory of the late Dr. Benjamin Rush, Professor of the Institutes and Practice of Medicine, and of Clinical Practice in the University of Pennsylvania. Delivered and published at the request of the Graduates and Students of Medicine, by William Staughton, D.D. Philadelphia.

### TO CORRESPONDENTS.

*Communications have been received from Mr. Rees,—A Regular Physician,—Mr. Whitford, &c. &c. &c. which will appear in our next.*

